# Neoadjuvant Chemoradiotherapy for Oesophageal Carcinoma: Real-World Data From Practice Beyond the Chemoradiotherapy for Oesophageal Cancer Followed by Surgery Study (CROSS) Trial

**DOI:** 10.7759/cureus.115499

**Published:** 2026-08-31

**Authors:** Ningthoujam Dinita Devi, Vikas K Jagtap, Rahul Mahawar, Onychia Synrem, Sumit Kumar, Anthialisha Nongkynrih, Batskhem Lyngkhoi, Caleb Harris, Sandeep Ghosh, Donboklang Lynser, Vandana Raphael

**Affiliations:** 1 Radiation Oncology, North Eastern Indira Gandhi Regional Institute of Health & Medical Sciences, Shillong, IND; 2 Radiation Oncology, North Eastern Indira Gandhi Regional Institute of Health and Medical Sciences, Shillong, IND; 3 Radiation Oncology, Midnapore Medical College and Hospital, Kolkata, IND; 4 Surgical Oncology, North Eastern Indira Gandhi Regional Institute of Health and Medical Sciences, Shillong, IND; 5 Radiology, North Eastern Indira Gandhi Regional Institute of Health and Medical Sciences, Shillong, IND; 6 Pathology, North Eastern Indira Gandhi Regional Institute of Health and Medical Sciences, Shillong, IND

**Keywords:** carcinoma (ca) oesophagus, cross protocol, neoadjuvant chemoradiotherapy, neoadjuvant radiation therapy, oesophageal cancer

## Abstract

Background

The chemoradiotherapy for oesophageal cancer followed by surgery study (CROSS) protocol, combining neoadjuvant chemoradiotherapy with surgical resection, is the standard of care for patients with locally advanced oesophageal cancer. Real-world patients often don't meet CROSS trial criteria. We evaluated if the trial's positive results apply to patients with large, ineligible lesions.

Materials & methods

In this retrospective analysis, we included patients with oesophageal cancer treated between November 2022 and November 2025 who received neoadjuvant chemotherapy plus radiotherapy (NACT+RT) at a dose of 41.4 Gray (Gy) delivered in 23 fractions, with concurrent weekly paclitaxel and carboplatin, followed by surgical resection. For study purposes, patients were divided into two groups according to whether they met the CROSS-eligibility criteria. The CROSS-ineligibility criteria were defined as patients with any of the following clinical features, i.e., T4a disease, tumor length >8 cm, or planning target volume length >16 cm.

Result

Out of 65 cases, 69.2% (n=45) of patients were CROSS-ineligible. Fifty-one cases completed the intended treatment of neoadjuvant chemoradiation followed by surgery. The pathological complete response (pCR) rate was 58.8% (n=30) in the total cohort population (n=51) who underwent NACT+RT followed by surgery. Out of 36 CROSS-ineligible patients, 55.6% (n=20) had a pCR compared to 66.6% (n=10) in CROSS-eligible cases (n=15). Fourteen patients did not undergo surgery after NACT+RT. At a median follow-up of 17 months, the one- and two-year progression-free survival (PFS) rates for the entire cohort were 80% and 68%, respectively. The one-year PFS rate was 90% for CROSS-eligible cases (n=20) versus 80% (p=0.7) for CROSS-ineligible cases (n=45). Similar rates for two-year PFS were 54% and 62% (p=0.7) for CROSS-eligible and CROSS-ineligible cases, respectively. In a subset analysis of the patients who underwent surgery (n=51), the one-year PFS rates were 93% versus 90% (p=0.75) for CROSS-eligible (n=15) and CROSS-ineligible cases (n=36), respectively. Similarly, the two-year PFS rates were 43% versus 70% (p=0.75) for CROSS-eligible (n=15) and CROSS-ineligible cases (n=36), respectively. The one- and two-year overall survival (OS) rates for the entire cohort were 93% and 77%, respectively. The one-year OS rates were 95% for CROSS-eligible cases (n=20) versus 93% (p=0.8) for CROSS-ineligible cases (n=45). Similar rates for two-year OS were 54% and 72% (p=0.8) for CROSS-eligible and CROSS-ineligible cases, respectively.

In a subset analysis of patients who underwent surgery (n=51), the one-year OS rates were 93% versus 92% (p=0.78) for CROSS-eligible (n=15) and CROSS-ineligible cases (n=36), respectively. Similarly, the two-year OS rates were 43% versus 70% (p=0.78) for CROSS-eligible (n=15) and CROSS-ineligible cases (n=36), respectively.

Conclusion

With proper patient selection, the CROSS regimen can be safely extended to CROSS-ineligible patients, demonstrating its safety without compromising survival. We propose that oesophageal cancers presenting with cT4a tumours, tumours measuring greater than 8 cm, and nodal stages N2 and N3 can be safely treated with neoadjuvant chemoradiotherapy followed by surgery, a finding that warrants confirmation in larger prospective studies.

## Introduction

Esophageal cancer is the 13th most commonly diagnosed cancer and the seventh leading cause of cancer death worldwide, with an estimated 494,000 new cases and 442,000 deaths in 2024 [1]. Adenocarcinoma and squamous cell carcinoma are the two most common histological findings, with unique epidemiological and clinical trends. The disease exhibits significant geographic variability, with the highest prevalence observed in East Asia, where nearly 90% of all diagnosed cases of oesophageal cancer are squamous cell variant [2]. In contrast, western populations have seen a significant increase in oesophageal adenocarcinoma, primarily affecting the distal oesophagus and gastro-oesophageal junction, owing partly to the rising rates of Barrett’s oesophagus and gastro- esophageal reflux disease [3]. Despite recent breakthroughs in diagnostic and therapeutic techniques, the disease has an aggressive clinical course, frequent late-stage presentation, and poor survival outcomes [4]. The overall five-year survival rate remains low, at less than 20%, comparable to the outcomes seen in other aggressive malignancies such as pancreatic cancer, hepatocellular carcinoma, and advanced lung cancer [5]. This dismal prognosis is partly due to the advanced and late stage at presentation, as most cases have been identified with locally progressed or metastatic cancer, at which point curative therapy choices are severely limited [6]. Early studies revealed that surgery as a solitary method resulted in five-year survival rates of only 25-30%, and was commonly followed by significant locoregional relapse and distant metastatic spread, even after apparently curative reactions with negative margins [7,8].

Over the last two decades, the management of oesophageal carcinoma has changed dramatically, from surgery alone to integrated multimodal methods that include systemic therapy, radiation, and surgical resection. This evolution reflects a better understanding of oesophageal cancer to be considered as a systemic disease rather than a primarily locoregional one, demanding coordinated therapy approaches. Meta-analysis of randomized controlled trials found that neoadjuvant chemoradiotherapy (NACT+RT) provides a significant survival advantage compared to surgery alone, particularly in cases with squamous cell histological variant [9]. These findings prompted the global development and implementation of several NACT+RT programs. The CROSS protocol, which combines preoperative radiation and chemotherapy followed by surgical intervention, has become the standard management option for resectable and locally advanced oesophageal cancer patients after demonstrating significant improvement in survival outcomes [10]. The CROSS trial included patients aged 75 years or younger with potentially curable oesophageal or gastroesophageal junction carcinomas measuring no more than 8 cm in length, adequate hematologic, renal, hepatic, and pulmonary function, a World Health Organization (WHO) performance status of 2 or less, no history of prior malignancy, and weight loss of less than 10%. When compared to surgery alone, this approach improved five-year overall survival by 13% among patients with potentially resectable locally advanced oesophageal carcinomas [10,11]. Nonetheless, the evidence supporting these outcomes in real-world clinical settings outside of controlled trial scenarios remains limited.

In real-world populations, patients with oesophageal cancer typically exhibit clinical features that exclude them from participating in the CROSS procedure. Our study is conducted to evaluate whether the favorable outcomes seen in the CROSS trial can be safely and successfully reproduced in a patient group with larger lesions, beyond the inclusion criteria of CROSS and representative of routine clinical practice.

## Materials and methods

In this retrospective analysis, patients with oesophageal cancer treated with neoadjuvant chemotherapy plus radiotherapy (NACT+RT) at our center between November 2022 and November 2025 were included. As per standard assessment, all patients were subjected to upper Gl (gastrointestinal) endoscopy, biopsy, and a contrast-enhanced computed tomography (CECT) scan of the thorax and abdomen, or a positron emission tomography (PET) scan prior to the start of treatment. All patients were staged using American Joint Committee on Cancer (AJCC) 8th edition staging criteria [12]. All patients were treated with 41.4 Gray (Gy) of external beam radiation therapy (EBRT) delivered in 23 fractions over five weeks using 3-dimensional conventional radiotherapy (3DCRT) or volumetric ARC therapy (VMAT). Only two cases were planned with 3DCRT (as decided by the medical physicist and clinician to reduce lung doses), and the rest were treated with the VMAT technique. Gross tumour volume (GTV) was contoured on the planning CT scan images, taking into account all the information available from pretreatment endoscopy, CT scan, or PET scan imaging. The GTV was defined as the gross primary tumour and the enlarged lymph nodes [13]. A supero-inferior margin of 3 cm (along the mucosa) and a circumferential margin of 0.5-1 cm (as per clinician's discretion) were added around the GTV to create the clinical target volume (CTV). The CTV was modified from anatomical barriers such as lung, heart, bones, and major vessels. A 0.7 cm margin was added to the CTV to create the planning target volume (PTV). The cranio-caudal length of the PTV for each case was measured on the planning CT scan using the measurement tool in the planning system. Dose volume constraints as per National Comprehensive Cancer Network (NCCN) guidelines were applied during treatment planning [14]. Daily image guidance with cone beam CT scan (CBCT) was performed for all patients during the radiation delivery. Patients also received concurrent chemotherapy with paclitaxel (50mg/m²) and carboplatin (AUC-2) on a weekly basis.

All patients were planned for surgical resection within six to eight weeks after chemoradiation. Patients underwent a radical esophagectomy performed via an open approach (i.e., transthoracic or transhiatal surgical resection) or a minimally invasive approach with locoregional node dissection involving mediastinal and upper abdominal lymph node regions. For study purposes, patients were divided into two groups according to whether they met the CROSS-eligibility criteria or not. CROSS ineligibility was defined by the presence of any of the following clinical features: T4a disease, tumor length >8 cm [as per Upper GI endoscopy (UGIE)], or planning target volume length >16 cm. The primary endpoint was the pathological complete response (PCR) rate, denoted by ypT0N0 after surgery, while the secondary endpoints included progression-free survival (PFS) and overall survival (OS) rates. Treatment-related toxicities were analyzed using common terminology criteria for adverse events (CTCAE) Version 5.0 [15]. All patients underwent UGIE and a CECT or PET scan four weeks after completion of NACT+RT. Routine UGIE was done every three months for patients on follow-up. CECT or PET scans were advised if there was any clinical suspicion of disease recurrence. PFS was defined as the time from the start of treatment to disease progression, and OS was defined as the time from the start of treatment to death or last follow-up. The Kaplan-Meier method was used to analyze survival, and differences were calculated using the log-rank test. The study protocol and all related procedures were reviewed and approved by the Institutional Ethics Committee (IEC).

## Results

Between November 2022 and November 2025, sixty-five patients with carcinoma of the oesophagus were planned for NACT+RT followed by surgery as per the CROSS protocol. Out of 65 cases, only 51 patients completed the planned treatment of NACT+RT followed by surgery. Fourteen patients did not undergo surgery after NACT+RT treatment (Figure 1).

**Figure 1:**
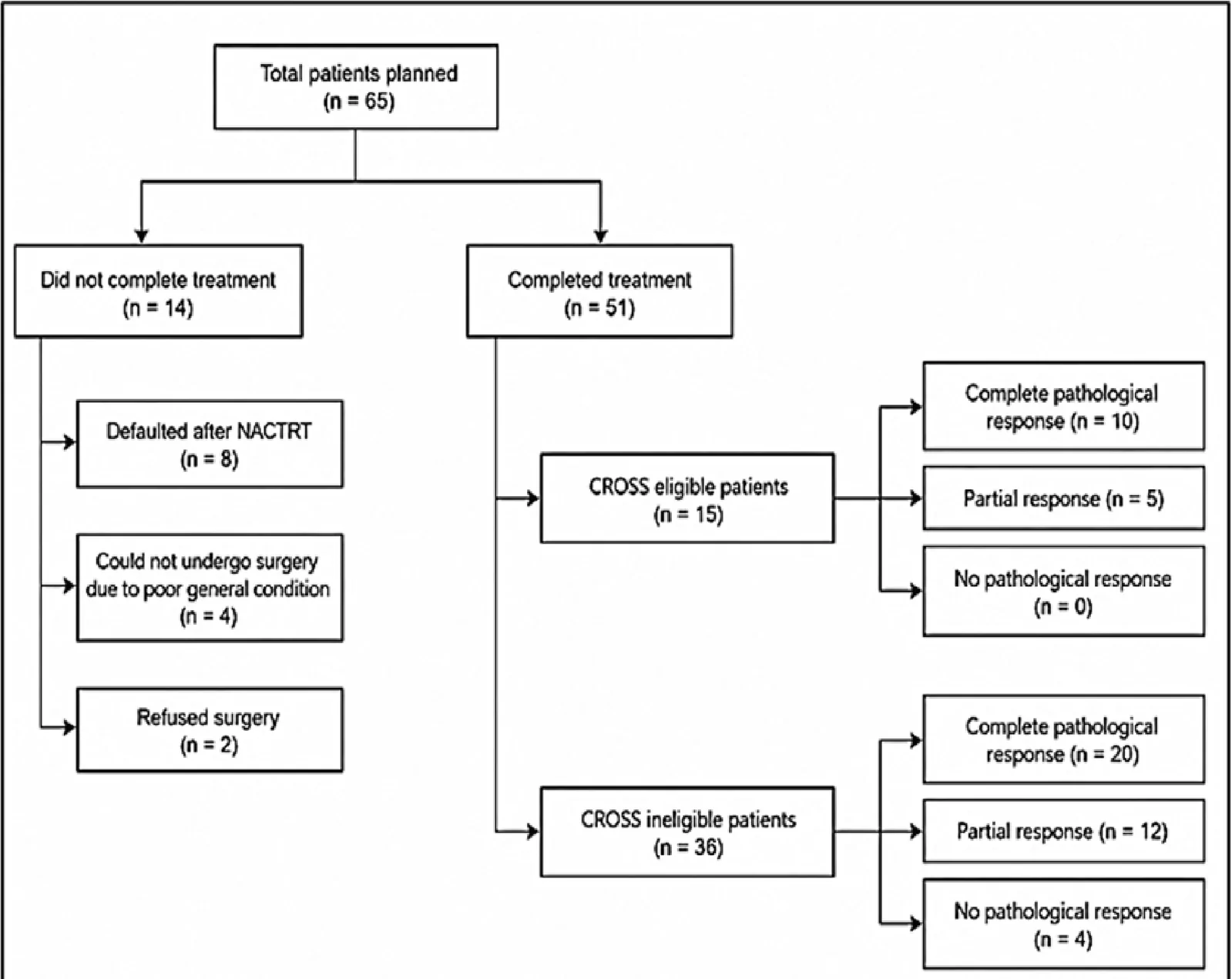
CONSORT diagram showing patient selection and inclusion in the study NACT+RT: neoadjuvant chemotherapy plus radiotherapy; CROSS: chemoradiotherapy for oesophageal cancer followed by surgery study.

The patients’ demographic profile and clinical details are shown in Table 1.

**Table 1:** Baseline demographic and clinical parameters of patients (N=65) cm: centimeter, cT: clinical tumour stage, cN: clinical nodal stage; CROSS: chemoradiotherapy for oesophageal cancer followed by surgery study.

Demographic and Clinical Parameters		Number (%)
Sex	Male	47 (72.3)
	Female	18 (27.7)
Age median (range)	56 years (35–76 years)	-
Performance status	0	0
	1	52 (80)
	2	13 (20)
Histology	Squamous cell carcinoma	65 (100)
	Adenocarcinoma	0 (0)
Clinical T (Tumour) stage	cT3	57 (87.7)
	cT4a	8 (12.3)
Clinical N (Nodal) stage	cN0	21 (32.3)
	cN1	19 (29.3)
	cN2	23 (35.4)
	cN3	2 (3)
CROSS eligible	Yes	20 (30.8)
	No	45 (69.2)
Tumour length median (range)	6 cm (3–13 cm)	-

All patients in our analysis had advanced T3 and T4 tumours, and none had T1 or T2 stage tumours. Overall, 69.2% of the patients (n=45) did not match the CROSS-eligibility criteria as per study definition. Out of 65 cases, 20% had tumor length more than 8 cm (n=13), T4a stage disease was recorded in eight cases (12.3%), and PTV length > 16 cm was seen in 42 cases (64.6%) (Figure 2).

**Figure 2:**
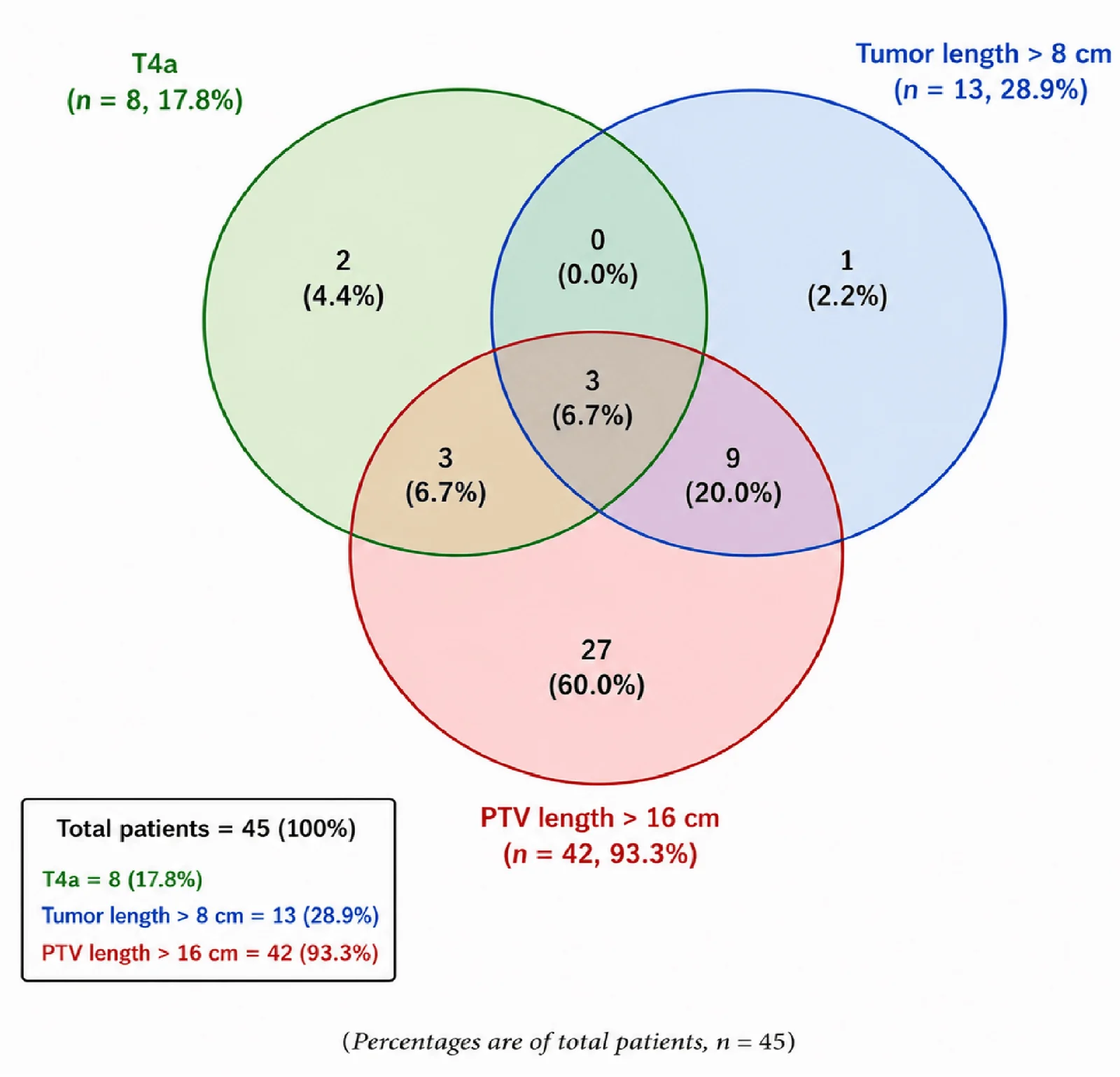
Venn diagram with Illustration of CROSS-ineligibility amongst the study population CROSS: chemoradiotherapy for oesophageal cancer followed by surgery study; PTV: planning target volume.

All cases completed the planned treatment of radiation and chemotherapy. With respect to concurrent weekly chemotherapy, 30.7% of patients (n=20) received five cycles, while 40% (n=26) completed four cycles of weekly paclitaxel and carboplatin chemotherapy regimen. The average planning target volume length was 18.4 cm, ranging from 11 to 29 cm. R0 resection (No tumor left behind, confirmed by microscope in the final histopathology report) was achieved in 51 patients (100%) who underwent surgery. Out of 51 cases who underwent surgery after NACT+RT, a complete pathological response (pCR) defined as ypT0N0, was achieved in 30 patients (58.8%). Out of thirty-six CROSS-ineligible patients, 55.6% (n=20) had pCR compared to 66.6% (n-10) in CROSS-eligible cases (n=15). The median number of lymph nodes dissected was 37 (range, 15-66). No patient experienced treatment-related toxicity of grade 3 or higher. After a median follow-up of 17 months, the one-year and two-year progression-free survival (PFS) rates for the entire cohort were 80% and 68%, respectively (Figure 3).

**Figure 3:**
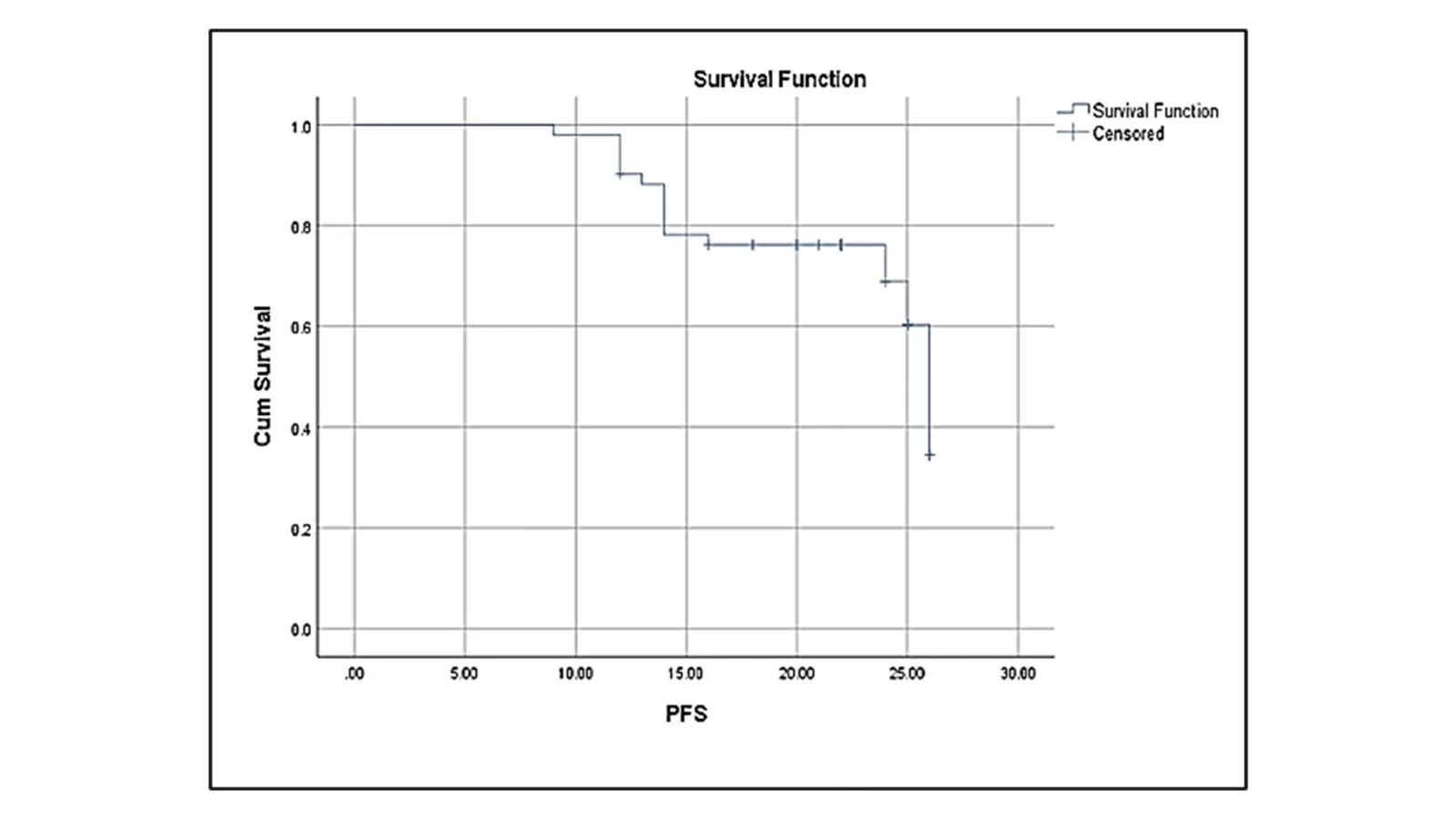
Kaplan-Meier curve of PFS estimate for entire cohort (N=65) PFS: progression free survival.

The PFS and OS rates for the study population and subset analysis of CROSS-eligible & CROSS-ineligible groups are shown below (Tables 2, 3 and Figure 4).

**Table 2:** PFS and OS rates as per study groups CI: confidence interval; NACT+RT: neoadjuvant chemotherapy plus radiotherapy; PFS: progression free survival; OS: overall survival

Survival	Year	Entire study population (n=65)	95 % CI (In months)	Patients who underwent surgery after NACT+RT (n=51)	95 % CI ( In months)
PFS	1 year	80%	21.2-24.3	90%	21.2-24.3
	2 years	68%	21.2-24.3	68%	21.2-24.3
OS	1 year	93%	21.4-24.3	92%	21.4-24.
	2 years	77%	21.4-24.3	68%	21.4-24.4

**Table 3:** Subset analysis - PFS and OS rates for patients CI: confidence interval; NACT+RT: neoadjuvant chemotherapy plus radiotherapy; PFS: progression free survival; OS: overall survival

		Entire study population (n=65)					Patients who underwent surgery after NACT+RT (n=51)				
Survival	Year	Cross eligible (n=20)	95 % CI (In months)	Cross ineligible (n=45)	95 % CI (In months)	P value	Cross eligible (n=15)	95 % CI (In months)	Cross Ineligible (n=36)	95 % CI (In months)	P value
PFS	1 year	90%	20.3-25.5	80%	20.8-24.5	P = 0.7	93%	20.6-25.7	90%	20.6-24.4	P = 0.75
	2 years	54%	20.3-25.5)	62%	20.8-24.5	P = 0.7	43%	20.6-25.7	70%	20.6-24.4	P = 0.75
OS	1 year	95%	20-25.5	93%	21.1-24.6	P = 0.8	93%	20.6-25.7	92%	20.8-24.5	P = 0.78
	2 years	54%	20-25.5	72%	21.1-24.6	P = 0.8	43%	20.6-25.7	70%	20.8-24.5	P = 0.78

**Figure 4:**
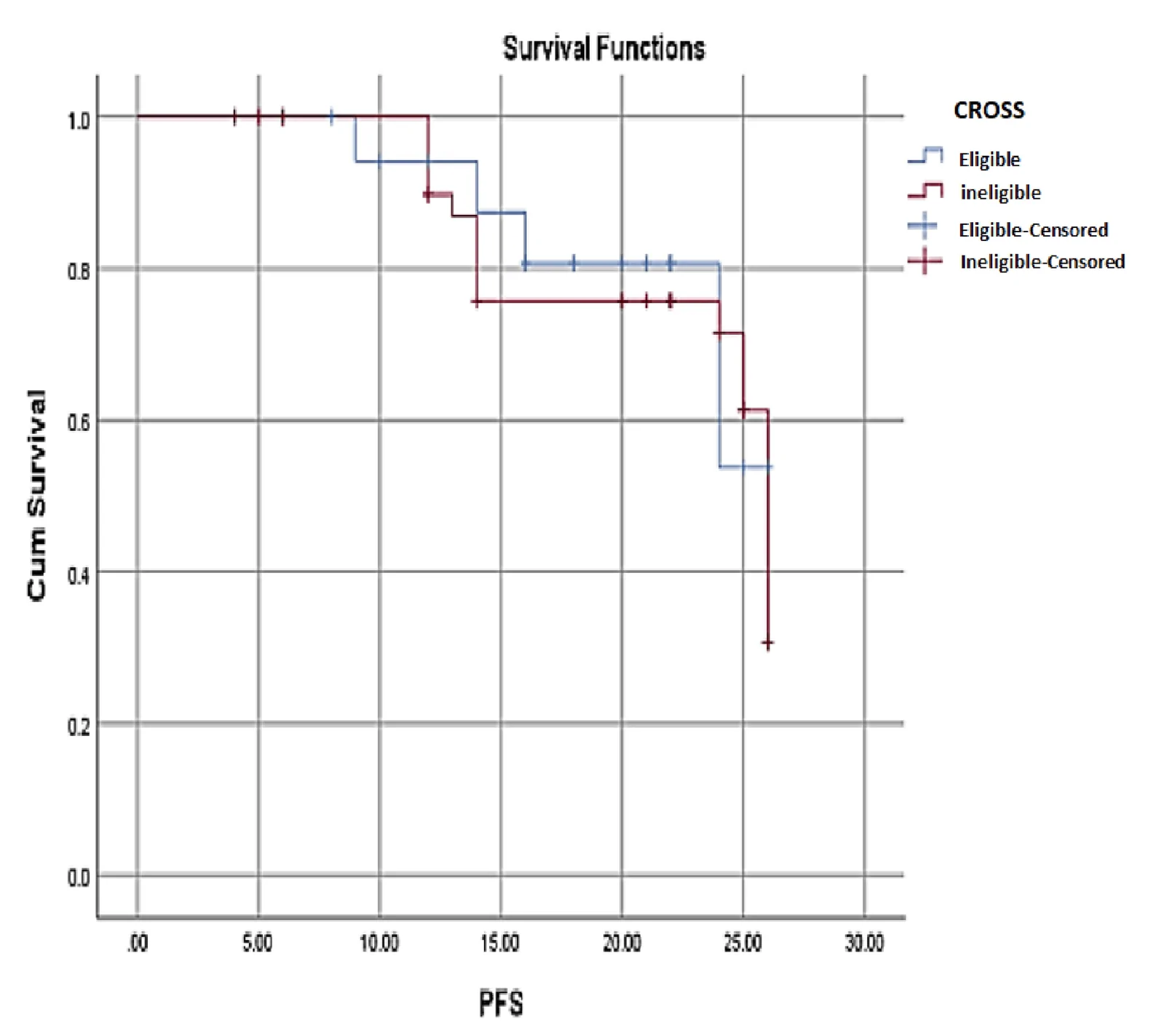
Kaplan-Meier curves of PFS estimates for CROSS-eligible (Blue colour) and CROSS-ineligible (Red Color) patients CROSS: chemoradiotherapy for oesophageal cancer followed by surgery study; PFS: progression free survival

## Discussion

The pathological complete response and PFS observed in the present study were comparable with those reported in the original CROSS trial. However, considering the differences in study design and patient selection criteria, comparisons should be interpreted with caution [10]. In this study, the one-year and two-year PFS rates in the CROSS-ineligible group were 80% and 62%, respectively, which were higher than the equivalent PFS outcomes of 71% and 60% reported for the neoadjuvant chemoradiotherapy arm of the CROSS trial [10]. Furthermore, surgical outcomes and progression-free survival rates were comparable across patients who met the CROSS-eligibility requirements and those who did not. Our results show that the benefits of neoadjuvant chemoradiotherapy can be extended beyond the eligibility criteria defined in the CROSS trial. In routine practice, we often encounter patients with T4a disease and advanced nodal stages who can be safely considered for neoadjuvant chemoradiotherapy based on our results, without compromising response rates or survival. We did not include N2 and N3 disease as CROSS trial ineligibility criteria in our study because patients in the original CROSS trial were included according to the AJCC 6th Edition [16]. Under the AJCC 6th Edition, N1 was defined as the involvement of regional lymph nodes based on the location of the primary tumor, without any N2 or N3 criteria. In contrast, under the AJCC 8th Edition, N1, N2, and N3 are defined as metastasis in one or two, three to six, and seven or more regional lymph nodes, respectively [12]. In addition, a PTV length greater than 16 cm was selected as an exclusion criterion in our study because the original CROSS trial added proximal and distal margins of 4 cm beyond the GTV [17]. Considering the maximum tumour length criterion of 8 cm in the CROSS trial, our assumption of a 16 cm PTV length appears justifiable.

In a recent study of 161 patients with oesophageal cancer, the results of those who met the initial CROSS-eligibility criteria were compared with those who met enhanced requirements [18]. Broadening eligibility did not result in higher neoadjuvant chemoradiotherapy-related toxicity, postoperative morbidity, or postoperative mortality; however, overall survival rates were slightly higher in the CROSS-eligible groups [18]. Our data are also supported by a similar report from our region suggesting the CROSS-protocol implementation outside clinical trial settings with expanded eligibility criteria [19]. In another study, there was no significant difference in the pCR rate, but it was associated with an increase in postoperative mortality and morbidity rates [20]. These findings suggest that the CROSS regimen maintains clinical efficacy outside of controlled studies, implying that it can be used in routine treatment in carefully selected patients with advanced oesophageal cancer. An R0 resection was obtained in 100% of patients in the current cohort, which is comparable to the percentage reported in the CROSS trial's neoadjuvant chemoradiotherapy arm [10]. Pathological complete response, as defined by the absence of residual tumour in both the primary lesion and regional lymph nodes (ypT0N0), was achieved in 55.6% of the CROSS-ineligible patients and 66.7% of the CROSS-eligible cases in our data, which is slightly higher than the reported pCR rate of 49% in the CROSS study's squamous cell carcinoma subgroup [21]. Even though only 70% of our patients received four to five cycles of weekly concurrent chemotherapy compared to 95% of cases receiving all five cycles of weekly chemotherapy in the CROSS trial, our results suggest the benefit of the NACT+RT approach in routine clinical practice where the patients may not always complete the full CROSS protocol.

It is also pertinent to note that none of our cases were diagnosed with adenocarcinoma histology. Prevalent consumption of betel nut with slaked lime with concomitant use of tobacco and alcohol in our region could be one of the reasons for this finding [22]. In another study, Shen J et al. demonstrated a pathological complete response rate of 43.2% in patients treated with preoperative chemoradiation followed by surgery, which is lower than our findings [23]. Similarly, the JCOG-Next trial demonstrated a pCR of 38.5% and R0 resection rate of 98% in the Neo CF+RT group, which is also lower than our findings [24]. The primary limitations of this study are its retrospective nature and small sample size. The relatively short median follow-up of 17 months may limit the detection of locoregional relapse and distant metastatic progression, especially given the CROSS-trial findings, which show that the majority of these events occur within 24 and 30 months, respectively [10]. A prospective approach would have allowed for more comprehensive and systematic assessment of treatment-related toxicity. Despite these limitations, the present findings have significant clinical implications. The ability to achieve outcomes comparable to those reported in the CROSS trial in a more diverse patient population supports the wider adoption of neoadjuvant chemoradiotherapy as a standard treatment approach in patients who would not have met the original trial eligibility criteria. Jiwei Wu et. al. demonstrated that achieving a pathological complete response (pCR) after neoadjuvant therapy is associated with improved survival in patients with oesophageal squamous cell cancer [25]. This finding is particularly relevant given the fact that the majority of oesophageal cancer patients in our clinics have advanced-stage disease, and Multidisciplinary Tumour Board (MDT) discussions could make treatment decisions easier and positively impact survival [26]. Curative-intent multimodal therapy with NACT+RT followed by surgery is a potentially life-extending treatment option in such cases.

## Conclusions

Our analysis provides real-world evidence demonstrating the feasibility and treatment effectiveness of the CROSS protocol in a patient population that is representative of routine clinical practice. With proper patient selection, the CROSS regimen can be safely extended to CROSS-ineligible patients, demonstrating its safety without compromising survival. We propose that oesophageal cancers presenting with cT4a tumours, tumours measuring greater than 8 cm, and nodal stages N2 and N3 can be safely treated with neoadjuvant chemoradiotherapy followed by surgery, a finding that warrants confirmation in larger prospective studies.
